# Coaching leadership exercised by nurses in the context of pre-hospital care: an analytical observational study

**DOI:** 10.1590/0034-7167-2024-0635

**Published:** 2025-12-08

**Authors:** Letícia Santiago Utsch, Fernanda Oliveira Santos, Katharina Júlia Lacerda Oliveira, Lara Campos Silva Santos, Bruna Franco Massa, Chennyfer Dobbins Abi Rached, André Almeida de Moura

**Affiliations:** I Universidade Federal de Minas Gerais. Belo Horizonte, Minas Gerais, Brazil; II Universidade de São Paulo. São Paulo, São Paulo, Brazil

**Keywords:** Leadership, Nurses, Emergencies, Emergency Medical Services, Nursing Staff., Liderazgo, Enfermeras y Enfermeros, Urgencias Médicas, Servicios Médicos de Urgencia, Personal de Enfermería.

## Abstract

**Objectives:**

to analyze the correlation between coaching leadership exercised by pre-hospital care nurses and sociodemographic and professional aspects.

**Methods:**

an observational, analytical, cross-sectional study, carried out in mobile and fixed pre-hospital care units in Minas Gerais. A total of 79 nurses answered the questionnaires “Characterization of participants with sociodemographic/professional data” and “Nurse Self-Perception Questionnaire in Leadership Exercise Questionnaire”.

**Results:**

nurses perceive themselves as exercising coaching leadership (score 83.89). Communication was the dimension with the highest mean of the leadership instrument. Being a cis male nurse influenced the support of the team in this context. Communication was correlated with having a fixed-term contract and not having another employment relationship.

**Conclusions:**

nurses perceive coaching leadership in nursing practice, and men may be more likely to adopt leadership styles that emphasize active support to the team with a focus on results.

## RESUMEN

**Objetivos:**

analizar la correlación entre el liderazgo coach ejercido por enfermeras de atención prehospitalaria y aspectos sociodemográficos y profesionales.

**Métodos:**

estudio observacional, analítico, transversal, realizado en unidades de atención prehospitalaria móviles y fijas de Minas Gerais. Un total de 79 enfermeras respondieron los cuestionarios “Caracterización de los participantes con datos sociodemográficos/profesionales” y “Cuestionario de Autopercepción de Enfermeras en el Ejercicio del Liderazgo”.

**Resultados:**

las enfermeras se perciben a sí mismas como personas que ejercen un liderazgo de coaching (puntuación 83,89). La comunicación fue la dimensión con mayor promedio del instrumento de liderazgo. Ser enfermero cis influyó en el apoyo del equipo en este contexto. La comunicación estaba correlacionada con un contrato de duración determinada y ninguna otra relación laboral.

**Conclusiones:**

las enfermeras perciben la práctica del coaching de liderazgo en la práctica de enfermería y los hombres pueden ser más propensos a adoptar estilos de liderazgo que enfaticen el apoyo activo al equipo con un enfoque en los resultados.

## INTRODUCTION

The role of nurses in emergency services is highly relevant and indispensable, given that these professionals are in direct contact with patients, from the very first moment (at the scene), via the Mobile Emergency Care Service (In Portuguese, *Serviço de Atendimento Móvel de Urgência* - SAMU), through fixed pre-hospital care, hospital services, and until patient are discharged. All of these care scenarios require nurses to have specific attitudes, skills, technical-scientific knowledge, and ethical aspects^(1)^.

In this same sense, it is worth noting that work in emergency rooms involves a lot of dynamism, given that the healthcare team involved is the one that drives the functional response capacity. Nurses play essential roles in this scenario. Given this, leadership is a fundamental skill, because it is from and through leadership that synchrony in teamwork, quality care, and harm reduction are achieved, thus enabling better results for patients^(2)^.

Additionally, an integrative review showed that leadership is a skill that, for its effective use, this professional must keep up to date and make use of other skills. Furthermore, there are long-standing challenges in emergency care worldwide, such as workforce shortages and work overload; an aging population with complex comorbidities and overcrowding in emergency departments. All of these factors continue to compromise the ability to provide timely care and to exercise leadership. Another important point is that nurses working in this context need greater recognition and appreciation^(3)^.

Since pre-hospital care (PHC) - both mobile and fixed - is a service with complex and stressful activities, physical and emotional exhaustion can occur, making it extremely important to assess the leadership of nurses in this context. In view of this, a study conducted in the countryside of São Paulo with the nursing team showed that the leader has a fundamental role in elucidating the values of those led, encouraging them to develop their own qualities, and consequently promoting a healthy practice environment within the nursing team^(4)^.

Nurses are notoriously crucial in health systems and services, with the most comprehensive challenges possible. To this end, nurses, faced with difficulties in carrying out their functions, can use leadership models or styles to contribute to the exercise of their professional practice^(5)^.

Among the new leadership approaches, coaching leadership stands out, which is supported by situational leadership and based on the coaching process, and is conceptualized as a competency in which a leader seeks to influence the team to achieve objectives, while enabling the development of skills, knowledge and attitudes of those led. This leadership model is based on the “Communication”, “Give and receive feedback”, “Delegate power and exert influence” and “Support the team so that the organizational results are reached” dimensions, which constitute elements or competencies necessary for the exercise of nurse leader^(2,6,7)^.

It is essential that institutions become organizational learning systems, in which teamwork is encouraged and where people can benefit from autonomy and self-realization. In the absence of this, people feel limited, making it essential to liberate, guide and encourage them. Thus, the coaching leadership process constitutes a fundamental competency for self-correction of behavior and learning within the organization, and the role of coaches translates into an inevitable element in the institution^(6)^.

Building relationships and maintaining positive relationships in the nursing workplace helps keep staff engaged and motivated. In this way, leadership coaching provides tools that can be used to support and improve team performance^(8)^. Based on this, it is worth highlighting that the only two national studies on the analysis of this leadership model in the PHC context show that nurses, when performing their coaching leadership effectively, promote positive transformations in their workspace, considering job satisfaction, thus enabling the provision of quality and safe care for the population assisted by this service^(2,9)^.

Furthermore, in one of these two studies, the relationship between this leadership model and sociodemographic and professional variables was also analyzed, with results demonstrating that, in the self-perception of coaching leadership of nurse coordinators with less time since graduation, a correlation was evident with the “Give and receive feedback” and “QUAPEEL total score” domains. These relationships indicate the need for investigations into the relationship between these variables, in order to propose strategies such as investment by health organizations in professionals with more time since graduation, in order to perceive the impact of this competency on the PHC nursing team, in the work environment and in the care provided to patients.

Recent studies, which were carried out in other contexts, but using this same leadership model, showed that the more the nurse exercises the coaching leadership dimensions in the hospital unit, the greater the perception of nursing professionals regarding the safety climate and team satisfaction^(10)^. This model is influenced by the characteristics of hospital organizations, but it may present possibilities for improvement as a competency for quality of patient care, in addition to establishing new ways of leading a team to achieve institutional goals^(7,11)^. In addition, it contributes to creating environments that are more favorable to professional practice and that Primary Health Care nurses make extensive use of the “Communication” dimension for this process^(12)^. These studies show that work is concentrated in the hospital context and with less expressiveness in practice environments of Primary Health Care and PHC nurses.

Thus, the need for new studies that identify the aspects that require improvement and discussion about coaching leadership is evident. Furthermore, the articles have pointed to the development of new investigations that seek to analyze the aspects that may be influencing the practice of this leadership model, such as sociodemographic and professional factors^(2)^ and that explore the leadership interfaces established by fixed and mobile pre-hospital nurses^(3)^. Therefore, the present research advances, in this sense, by answering the following question: what is the relationship between the perception of coaching leadership of PHC nurses (fixed and mobile) and sociodemographic and professional variables?

## OBJECTIVES

To analyze the correlation between coaching leadership exercised by nurses at mobile and fixed PHC and sociodemographic and professional aspects.

## METHODS

### Ethical aspects

This study was assessed and approved by the Research Ethics Committees of *Universidade Federal de Minas Gerais* and the Municipal Health Department of Belo Horizonte, in order to meet the determinations of Resolution 466/2012 of the Brazilian National Health Council. For data collection, it is reiterated that all participants signed an Informed Consent Form before the questionnaires were applied.

### Study design, period and location

This is an observational, analytical and cross-sectional study, aimed at examining the relationship between coaching leadership and sociodemographic and profession-related factors. This article followed the of STrengthening the Reporting of OBservational studies in Epidemiology recommendations to support the communication of the results shown^(13)^. The study setting was the Emergency Network (EN) of a city in the Metropolitan Region of the capital of the state of Minas Gerais. This EN consists of SAMU 192 and nine Emergency Care Units (ECUs). SAMU 192 and six ECUs from the metropolitan capital of Minas Gerais participated in the research. Three ECUs were not included because they did not respond to emails to collect data. Data collection took place between July and December 2023.

### Population, sample; inclusion and exclusion criteria

The total number of nurses in the units participating in the research, both in the care and management areas, totaled 258 professionals. Nurses who had been in the position for at least six months were included, in order to ensure that professionals had already adapted and, therefore, were able to analyze the leadership practice. Professionals in the aforementioned category who were on vacation, on leave or on sick leave during the data collection period were excluded.

Given these criteria, it is possible to point out that: 34 nurses were on vacation; ten were on medical leave; 39 professionals had not completed the minimum period of six months at the institution; and 21 professionals refused to participate in the research. Thus, 154 nurses were effectively eligible for the research. Based on this value, sample calculation was applied, adopting a 8% sampling error, a 95% confidence level and a more homogeneous distribution of the population (80/20), obtaining 61 participants. This was a convenience sample, and the researchers chose to deliver the questionnaires to all nurses who worked at SAMU and the six ECUs during the collection period. It is important to note that professionals who, after two attempts, were not located on their respective shifts and professionals who did not return the completed or blank questionnaires were considered as sample losses for the present study.

### Data instruments, collection and analysis

Two questionnaires were used, the first being constructed by the researchers themselves, not being validated by judges and corresponding to the characterization of participants, such as sociodemographic and professional data, whose information collected consisted of: age; sex; time since graduation; complementary training; type of employment relationship; time working in the unit; having another employment relationship; and having already taken over a nursing coordination/leadership position.

The third part of an instrument entitled Nurse Self-Perception Questionnaire in Leadership Exercise Questionnaire (In Portuguese, *Questionário de Autopercepção do Enfermeiro no Exercício da Liderança* - QUAPEEL) was applied, which contains items related to the skills and attitudes exercised by leaders in coaching leadership practice, taking into account the professional’s self-perception about coaching leadership. This instrument was developed and validated in Brazil and, in order to carry out this research, the author’s authorization to use the instrument was previously requested and granted^(6)^.

This questionnaire has 20 items, subdivided into four domains: Communication (items 1 to 5); Give and receive feedback (items 6 to 10); Delegate power and exert influence (items 11 to 15); and Support the team so that the organizational results are reached (items 16 to 20). For all items, there is a Likert scale arranged as follows: never (1); rarely (2); not always (3); almost always (4); and always (5). The “not applicable” (NA) refers to the item that a professional believes they do not develop, and in this sense, the value is assigned to zero. Thus, the general instrument score varies between 0 and 100, considering that the values closest to 0 correspond to the lowest perception of the practice of coaching leadership, and 100, to the highest perception of the practice of this type of leadership^(6)^.

The group, consisting of a research professor and four research students, was responsible for delivering the questionnaires to the subjects who agreed to participate in the research. To this end, the administrative and nursing coordinators of the units in question were contacted in advance so that the questionnaires could be delivered to the nurses in person as well as scheduling the collection of the instruments. It is worth noting that, in order to deliver the questionnaires, three attempts were made on different dates, in order to find the professionals on their respective shifts. After these three unsuccessful attempts, the population was considered lost.

The data collected through the questionnaires were double-entered into spreadsheets and compared, thus ensuring that there were no typing errors. Descriptive statistical analysis and correlation between variables were then performed. For this purpose, the statistical program R version 4.3.3 was used to process the data. Qualitative variables were summarized in simple and relative frequencies, using percentages. Quantitative variables were organized by mean, median, standard deviation, first and third quartile, and minimum and maximum values.

Initially, the Shapiro-Wilk normality test was used to assess the distribution of variables, while Pearson’s correlation was applied to analyze the correlation between quantitative variables, such as age, time since graduation and time in the institution, and coaching leadership variables. Likewise, the Wilcoxon-Mann-Whitney and Kruskal-Wallis statistical tests were used to analyze the level of significance between coaching leadership variables and sociodemographic and professional variables. A significance level of 5% was adopted for all statistical tests (α=0.05).

In addition to Pearson’s test, associations between variables present in the study were assessed according to the size of the correlation, positive or negative, and could therefore present: very high correlation (0.9 to 1.0); high correlation (0.7 to 0.89); moderate correlation (0.40 to 0.69); weak correlation (0.1 to 0.39); and insignificant correlation (0.0 to 0.3 or 0.0 to -0.3)^(14)^.

## RESULTS

At the end of data collection, a percentage of 48.17% (n=79) of participants was obtained. It is worth noting that the remaining professionals (n=75), i.e., 53 of them, did not return the envelopes with the questionnaires and, after two attempts, 22 of these professionals were not located due to changes in their respective shifts.

It was found that nurses were predominantly cis female (n=61; 77.22%), civil servants, i.e., governed by the statutory regime (n=35; 44.30%), workers with another employment relationship (n=40; 50.63%) and nursing assistants, as they had never held a nursing coordination/management position (n=49; 62.03%). Concerning training, a considerable number of professionals had completed a specialization (n=70; 88.61%); ten professionals had a master’s degree; only one nurse had a doctoral degree (1.27%); and just under half had completed a course complementary to their training (n=37; 46.84%). Regarding quantitative variables related to sociodemographic aspects or related to the profession, nurses had a mean age of 41.10 years (SD=8.12), 13.66 years of training in relation to the date of graduation (SD=7.32) and 5.95 years of employment in the unit (SD=6.35). Table 1 shows the distribution of the scores of the measures of nurses’ self-perception regarding the coaching leadership practice exercised by the nurses themselves in the PHC units.

**Table 1:** Self-perception scores of nurses exercised in pre-hospital care units, Belo Horizonte, Minas Gerais, Brazil, 2023 (N=79)

Domains	Mean	Standard deviation	Median	Minimum	Maximum
Communication	22.47	2.25	23	17	25
Give and receive feedback	21.72	2.60	22	13	25
Delegate power and exert influence	20.85	3.23	21	8	25
Support the team so that the organizational results are reached	18.85	4.80	20	5	25
QUAPEEL total score	83.89	10.44	84	55	100

Table 1 shows that nurses had a self-perception of the exercise of coaching leadership, considering the mean QUAPEEL total score, which obtained the value of 83.89 (SD=10.44), i.e., the mean of the scale is closer to 100 than to zero.

When correlations between coaching leadership and time since graduation, time working in the unit and age are carried out, it is found that there is no correlation (Table 2).

**Table 2:** Correlation between the dimensions of coaching leadership practice in the self-perception of nurses in pre-hospital care units and sociodemographic and professional quantitative variables, such as age, time since graduation and time in the unit, Belo Horizonte, Minas Gerais, Brazil, 2023 (N=79)

Domains	Time since graduation	Time in unit	Age
Communication	0.021^*^	-0.080	0.044
	0.856^**^	0.483	0.703
Give and receive feedback	0.053	-0.121	-0.042
	0.641	0.289	0.716
Delegate power and exert influence	0.097	-0.006	0.037
	0.397	0.962	0.746
Support the team so that the organizational results are reached	-0.070	-0.201	-0.117
	0.543	0.076	0.304
QUAPEEL total score	0.016	-0.141	-0.043
	0.891	0.214	0.704

* Correlação de Pearson;

** Valor de p.

From Table 3, it was found that coaching leadership presented a statistically significant difference for the male cis gender and the “Support the team so that the organizational results are achieved” dimension, i.e., male cis gender nurses have a greater perception in promoting support to the team (p=0.044); professionals whose type of contract is for a fixed period; the “Communication” dimension is exercised more presently (p=0.043); and not having another employment relationship influences the perception of the “Communication” dimension (p= <0.001).

**Table 3:** Mean scores of the Nurse Self-Perception Questionnaire in Leadership Exercise Questionnaire dimensions and total score of nurses working in pre-hospital care units, according to sociodemographic and professional qualitative variables, and the correlation between these variables, Belo Horizonte, Minas Gerais, Brazil, 2023 (N=79)

		Communication	Give and receive feedback	Delegate power and exert influence	Support the team so that the organizational results are reached	Nurse Self-Perception Questionnaire in Leadership Exercise Questionnaire total score
Sex	Female	22.28	21.61	20.62	18.38	82.89
	Male	23.11	22.11	21.61	20.44	87.28
*p* value^*^		0.070	0.436	0.304	0.044	0.086
Type of employment relationship	Consolidation of Labor Laws	22.17	21.33	20.11	18.83	82.44
	Statutory	22.09	21.29	20.91	17.69	81.97
	Fixed term contract	23.19	22.58	21.27	20.42	87.46
*p* value^*^		0.043	0.102	0.629	0.083	0.101
Has a complementary course	No	22.60	21.86	21.21	19.07	84.74
	Yes	22.32	21.57	20.43	18.60	82.92
*p* value^*^		0.523	0.606	0.384	0.764	0.443
Specialization	No	21.89	22.00	21.33	20.33	85.56
	Yes	22.54	21.69	20.79	18.66	83.67
*p* value^*^		0.638	0.969	0.810	0.670	0.613
Master’s degree	No	22.54	21.75	21.04	19.03	84.36
	Yes	22.00	21.50	19.50	17.60	80.60
*p* value^*^		0.271	0.959	0.317	0.245	0.309
Doctoral degree	No	22.51	21.72	20.82	18.81	83.86
	Yes	19.00	22.00	23.00	22.00	86.00
*p* value^*^		0.141	0.930	0.480	0.441	0.930
Other employment relationship	No	23.28	21.92	20.85	18.46	84.51
	Yes	21.68	21.53	20.85	19.23	83.28
*p* value^*^		< 0.001	0.523	0.650	0.887	0.533
Took over the role of nursing coordinator/leader	No	22.59	21.57	20.49	18.18	82.84
	Yes	22.27	21.97	21.43	19.93	85.60
*p* value^*^		0.377	0.879	0.368	0.205	0.256

* Wilcoxon-Mann;

** Whitney e Kruskal-Wallis.

## DISCUSSION

Leadership comprises a field of study in nursing over the years that explains, in part, its influence on the professional environment and organizational results, such as in the context of emergency^(2,3)^. The various leadership theories and models contribute to understanding, within the nursing work process, how relationships between leaders and subordinates occur^(15)^. Sociodemographic data and those related to the present research’s work are similar to those of research that analyzed the same population and instrument^(10,12)^ in the context of emergency care^(2)^. In this regard, it is worth highlighting that the performance of leadership in nursing is influenced by generational traits, since as Generation Z takes its place in the nursing workforce and in management, there has been an increase in potential for organizational changes and success, support for collaborative work, ease in shared learning, and the construction of strong and effective working relationships^(16)^.

It is noteworthy that coaching leadership was self-perceived by nurses in the mobile and fixed pre-hospital context with a mean (83.89), i.e., close to the value of 100, also evidenced in the pre-hospital study of the state of Goiás (mean of 80.91)^(2)^ and in the four studies developed in hospital environments, whose means of the instrument total score were 90.67^(7)^, 84.90^(10)^, 84.41^(17)^ and 82.59^(11)^. Given this result, coaching leadership is a powerful skill for performance in nursing work, given that it favors team development and improved professional performance, especially with regard to helping employees better understand themselves and their capabilities^(8)^.

Communication is an essential skill for successful nursing leadership, especially coaching leadership, because through effective communication, nurses can share their visions and influence their team. In this spectrum, among the instrument domains, the highest mean found was that corresponding to communication, with a mean of 22.47. Three other studies that applied QUAPEEL also identified communication as the main dimension of coaching leadership^(7,10,11)^. Supporting this finding, an integrative review suggests that resources invested in nurse leaders’ communication skills bring significant benefits in terms of team satisfaction, professional retention, quality of patient care and, ultimately, organizational success^(8,18)^.

Supporting the team to achieve results obtained the lowest value among the means of the instrument dimensions used in the research (mean of 18.85). Consistent with this finding, four other studies that analyzed coaching leadership in the hospital context and one in the PHC presented the lowest mean in this domain, compared to the others^(2,7,10,11,17)^. This confirms that it is still necessary for mobile and fixed PHC units to improve nurses’ skills and attitudes to encourage and support the team to achieve goals.

When considering the scenario of this study, it is clear that nurses play a fundamental role in the management of emergency care, making use of their potential, with emphasis on nursing team leadership and management^(3)^. No correlation was identified between study quantitative variables and QUAPEEL dimensions and total score. Similarly, a study carried out in the countryside of São Paulo also identified that age and time since training of respondents did not influence exemplary leadership practice assessment^(4)^.

In contrast to these findings, another study identified a negative correlation between “Give and receive feedback” and “time since graduation”, i.e., as the time since graduation increases, the nurses’ self-perception regarding the feedback process decreases. In this same study, there was also an inverse relationship between the coaching leadership “QUAPEEL total score” and the variable “time since graduation”, thus indicating that the longer the time since graduation, the lower the self-perception of coaching leadership practice^(2)^. It is noted that the time since graduation provides the leader with greater ease in detecting development needs, as well as training professionals to overcome the inseparable challenges of professional performance, through feedback that is significant and contributes to the high performance of the team and the achievement of organizational goals^(8)^.

Support the team so that the organizational results are showed a statistically significant difference for the cisgender male (p-value = 0.044). In this regard, it is worth noting that the literature on sociology, psychology and gender studies often discusses how gender socialization influences the assumption of leadership and support roles in work environments. Historically, men are encouraged in health organizations to assume leadership and support roles more than women, thus contributing to a special look at how men behave and are perceived in the nursing environment. In turn, women in nursing may face stereotypes that place them in positions of only support and direct care for patients, which can influence their interactions with the team^(19,20)^.

An integrative review indicates that men in nursing generally receive preferential treatment in career progression and are more accepted by managers, patients and other healthcare professionals in work processes^(20)^. Thus, considering the critical shortages affecting the profession worldwide, it is essential to promote work environments that encourage a more diverse nursing workforce, especially in terms of gender, race and ethnicity^(21)^.

Communication was influenced by qualitative variables, such as employment under a fixed-term contract and not having another employment relationship. These relationships can be justified by transformations in the nursing job market, as well as the progressive precariousness of employment relationships in the public sector that impact work processes and relationships and, consequently, the communication processes of health organizations^(22)^. Leadership coaching can play a role in developing effective communication, resolving conflicts and promoting a healthy work environment^(8)^.

There was no statistically significant difference between variables related to coaching leadership and having or not taken over a nursing coordination/management position. Considering this result, it is worth pointing out that a leader can influence and guide people without having formal authority over them (informal leadership). Moreover, coaching leadership is based on building trust and creating an environment of support and collaboration, while a leadership position often comes with formal authority and hierarchical responsibility^(8,23,24)^.

It is worth noting that, in this study, no relationship was found between dimensions, as well as the total QUAPEEL score, and having a specialization, master’s degree, doctoral degree or complementary course. Coaching leadership is an indispensable skill for achieving a healthy work environment, which results in patient safety, high job satisfaction and reduced staff turnover rates^(8)^. In this same sense, it is worth mentioning a study developed in the context of emergency, in the south of Minas Gerais, which identified that, in nurses’ perception, knowledge, professional experience and the ability to relate are crucial for professional success and for good results in care for service users^(25)^.

Supporting this finding, a study showed that, for the development of leadership, strategies are necessary that encompass the search for knowledge (such as postgraduate courses and complementary training), improvement of skills and adoption of positive attitudes, thus constituting essential elements for adequate performance of nurses in the exercise of their professional practice^(15)^. It is worth highlighting the great challenge of nursing leaders in the current scenario regarding the development of leadership, through involvement, team empathy and ability to influence and act enthusiastically^(26)^. Finally, although the relationship between experience in nursing coordination and leadership positions and the coaching leadership domains was not evidenced, a review indicates that coaching behaviors, such as feedback and open communication, can influence professionals’ organizational identification and well-being in the workplace^(8)^.

### Study limitations

A limitation of this study is the fact that it was not possible to obtain responses from all nurses in the units investigated due to the change of shifts. Although this difficulty was observed, the magnitude and innovative nature of work are ensured by the fact that it was possible to analyze both the context of mobile PHC, as SAMU, and ECUs. Another limitation is the non-validity of the instrument for characterizing the research participants, but this fact does not diminish the findings of the research.

### Contributions to health, nursing or public policy

Even in view of these limitations, this study is relevant because it has practical implications for the management and organization of mobile and fixed PHC services, especially with regard to nursing team management regarding factors that influence the leadership of these professionals. Therefore, managers must encourage and promote adequate conditions so that nurses can legitimately exercise their leadership, with due recognition.

## CONCLUSIONS

Through the findings of this study, nurses in the analyzed units had a self-perception of coaching leadership. The “Communication” dimension obtained the highest mean compared to the others, which demonstrates the relevance of this item for leadership practice by nurses in mobile and fixed pre-hospital units. It was found that coaching leadership was influenced by some sociodemographic or profession-related variables. A correlation was found between cis-male gender and the “Support the team so that the organizational results are achieved” dimension, in the same way that the “Communication” dimension was related to “fixed-term contract” and “not having another employment relationship” qualitative variables. No statistically significant differences or correlations were found between the dimensions and the total QUAPEEL score with quantitative variables. The study results point to possibilities for improvements, new ways to lead the team, in order to achieve the goals established in the mobile and fixed pre-hospital context.

## Data Availability

The research data are available within the article.
