# Supplementary material for: Coaching leadership exercised by nurses in the context of pre-hospital care: an analytical observational study
Source: Rev Bras Enferm. 2025 Dec 8;78(5):e20240635. doi: 10.1590/0034-7167-2024-0635 (PMC12707529; doi:10.1590/0034-7167-2024-0635)
Supplement: Translation

# Translation: Liderança *coaching* exercida pelos enfermeiros no contexto do atendimento pré-hospitalar: estudo observacional analítico

**DOI:** 10.1590/0034-7167-2024-0635pt

**Authors:** Letícia Santiago Utsch, Fernanda Oliveira Santos, Katharina Júlia Lacerda Oliveira, Lara Campos Silva Santos, Bruna Franco Massa, Chennyfer Dobbins Abi Rached, André Almeida de Moura

**Affiliations:** I Universidade Federal de Minas Gerais. Belo Horizonte, Minas Gerais, Brasil; II Universidade de São Paulo. São Paulo, São Paulo, Brasil

**Keywords:** Descritores: Liderança, Enfermeiras e Enfermeiros, Emergências, Serviços Médicos de Emergência, Recursos Humanos de Enfermagem.

## Abstract

**Objetivos:**

analisar a correlação entre a liderança *coaching* exercida pelos enfermeiros do atendimento pré-hospitalar e aspectos sociodemográficos e profissionais.

**Métodos:**

estudo observacional, analítico, transversal, realizado em unidades do atendimento pré-hospitalar móvel e fixo em Minas Gerais. Um total de 79 enfermeiros responderam aos questionários “Caracterização dos participantes com dados sociodemográficos/profissionais” e “Questionário de Autopercepção do Enfermeiro no Exercício da Liderança”.

**Resultados:**

os enfermeiros percebem-se no exercício da liderança *coaching* (escore 83,89). Comunicação foi a dimensão com maior média do instrumento de liderança. Ser enfermeiro do gênero cis masculino influenciou o apoio da equipe desse contexto. Comunicação apresentou correlação com o vínculo de contrato por tempo determinado e não possuir outro vínculo empregatício.

**Conclusões:**

enfermeiros percebem a prática da liderança *coaching* no exercício da enfermagem, sendo que homens podem ser mais propensos a adotar estilos de liderança que enfatizam o apoio ativo à equipe com foco em resultados.

## INTRODUÇÃO

A atuação do enfermeiro no serviço de urgência e emergência é de grande relevância e indispensável, haja vista que esse profissional está diretamente em contato com o paciente, desde o primeiro momento (no local da cena), através do Serviço de Atendimento Móvel de Urgência (SAMU), perpassando o pré-hospitalar fixo, serviço hospitalar, até a alta do paciente. Todos esses cenários de cuidado demandam do enfermeiro atitudes, habilidades, conhecimentos técnico-científicos, além dos aspectos éticos^(1)^.

Nesse mesmo sentido, cabe apontar que o trabalho na urgência envolve muito dinamismo, tendo em vista que a equipe de saúde envolvida é que conduz a capacidade funcional de resposta. Os enfermeiros desempenham papéis imprescindíveis nesse cenário. Diante disso, a liderança é uma competência fundamental. Porquanto, é a partir e por intermédio dela que se obtêm a sincronia do trabalho em equipe, um atendimento de qualidade e a redução dos danos, possibilitando, assim, melhores resultados para o paciente^(2)^.

Complementarmente, revisão integrativa evidenciou que a liderança é uma competência que, para sua efetiva utilização, o profissional deve se manter atualizado e fazer uso de outras competências. Ademais, existem desafios duradouros no atendimento de emergência em todo o mundo, como a escassez de mão de obra e sobrecarga de trabalho, uma população envelhecida e com comorbidades complexas, e superlotação das unidades de emergências. Todos esses fatores continuam a comprometer a capacidade de fornecer atendimento oportuno, assim como de exercer a liderança. Outro ponto de destaque é que os enfermeiros atuantes nesse contexto necessitam de maior reconhecimento e valorização^(3)^.

Por ser o atendimento pré-hospitalar (APH) - tanto o móvel quanto o fixo - um serviço de atividades complexas e estressantes, pode ocorrer um desgaste físico e emocional proporcionalmente intenso, sendo de grande relevância, portanto, avaliar a liderança dos enfermeiros nesse contexto. Diante disso, estudo desenvolvido no interior de São Paulo com a equipe de enfermagem mostrou que o líder tem um papel primordial de elucidar os valores dos liderados, incentivando-os a desenvolver suas próprias qualidades, promovendo, consequentemente, ambiente de prática saudável na equipe de enfermagem^(4)^.

Os enfermeiros, notoriamente, desempenham papel crucial nos sistemas e serviços de saúde, com os desafios mais abrangentes possíveis. Para tanto, os enfermeiros, diante das dificuldades para o desenvolvimento de suas funções, podem se valer de modelos ou estilos de liderança para contribuir para o exercício da sua prática profissional^(5)^.

Entre as novas abordagens de liderança, destaca-se a liderança *coaching*, que está amparada na liderança situacional e baseada no processo *coaching*, e conceitua-se como uma competência a qual o líder busca influenciar a equipe para alcance dos objetivos, ao mesmo passo que possibilita o desenvolvimento de habilidades, conhecimentos e atitudes dos liderados. Esse modelo de liderança se baseia nas dimensões “Comunicação”, “Dar e receber *feedback*”, “Dar poder e exercer influência” e “Apoiar a equipe para alcance de resultados”, que constituem elementos ou competências necessários para o exercício do enfermeiro líder^(2,6,7)^.

É essencial que as instituições se tornem sistemas de aprendizagem organizacional, nas quais o trabalho em equipe seja estimulado e onde as pessoas possam se valer de autonomia e autorrealização. Na ausência disso, as pessoas percebem-se limitadas, tornando-se imprescindível liberá-las, conduzi-las e incentivá-las. Assim, o processo da liderança *coaching* constitui uma competência primordial para a autocorreção do comportamento e aprendizado dentro da organização, e a figura dos *coaches* se traduz como elemento inevitável à instituição^(6)^.

A construção de relacionamentos e a manutenção de relações positivas no local de trabalho dos enfermeiros ajudam a manter os profissionais engajados e motivados. Dessa forma, a liderança *coaching* fornece ferramentas que podem ser utilizadas para o apoio e desempenho dos membros da equipe^(8)^. Baseado nisso, ressalta-se que os dois únicos trabalhos nacionais acerca da análise desse modelo de liderança no contexto APH mostram que os enfermeiros, ao desempenharem sua liderança *coaching* de forma eficaz, promovem transformações positivas no seu espaço de trabalho, considerando-se a satisfação no trabalho, possibilitando, assim, a prestação de cuidado com qualidade e segurança para a população atendida por esse serviço^(2,9)^.

Ainda, em um desses dois trabalhos, também foi analisada a relação entre esse modelo de liderança e as variáveis sociodemográficas e profissionais, tendo os resultados demonstrado que, na autopercepção da liderança *coaching* dos enfermeiros coordenadores com menor tempo de formação, evidenciou-se uma correlação com os domínios “Dar e receber *feedback*” e “Escore total do QUAPEEL”. Essas relações sinalizam a necessidade de investigações acerca da relação entre essas variáveis, de maneira a propor estratégias como o investimento das organizações de saúde para os profissionais com maior tempo de formação, de maneira a perceberam o impacto dessa competência na equipe de enfermagem do serviço pré-hospitalar, no ambiente de trabalho e no cuidado prestado ao paciente.

Estudos recentes, que foram realizados em outros contextos, porém utilizando esse mesmo modelo de liderança, evidenciaram que quanto mais o enfermeiro exercer as dimensões da liderança *coaching* na unidade hospitalar, maior a percepção dos profissionais de enfermagem quanto ao clima de segurança e à satisfação da equipe^(10)^. Esse modelo sofre influências das características das organizações hospitalares, no entanto pode apresentar possibilidades de melhoria como competência para qualidade de assistência ao paciente, além de estabelecer novas maneiras de liderar uma equipe para o alcance das metas institucionais^(7,11)^. Além disso, contribui para que haja ambientes mais favoráveis à prática profissional e que o enfermeiro da Atenção Primária à Saúde utilize bastante a dimensão “Comunicação” para esse processo^(12)^. Observa-se, a partir desses estudos, a concentração de trabalhos no contexto hospitalar e com menor expressividade nos ambientes de prática do enfermeiro da Atenção Primária à Saúde e do APH.

Assim, fica evidente a necessidade de novos estudos que identifiquem os aspectos que requerem aprimoramento e discussão acerca da liderança *coaching*. Ademais, os artigos têm apontado para o desenvolvimento de novas investigações que busquem analisar os aspectos que possam estar influenciando a prática desse modelo de liderança, a exemplo dos fatores sociodemográficos e profissionais^(2)^ e que explorem as interfaces da liderança estabelecidas pelos enfermeiros do pré-hospitalar fixo e móvel^(3)^. Portanto, a presente pesquisa avança, nesse sentido, ao responder à seguinte pergunta: qual a relação entre a percepção da liderança *coaching* dos enfermeiros do APH (fixo e móvel) e as variáveis sociodemográficas e profissionais?

## OBJETIVOS

Analisar a correlação entre a liderança *coaching* exercida pelos enfermeiros do APH móvel e fixo e aspectos sociodemográficos e profissionais.

## MÉTODOS

### Aspectos éticos

O presente estudo foi apreciado e aprovado pelos Comitês de Ética em Pesquisa da Universidade Federal de Minas Gerais e da Secretaria Municipal de Saúde de Belo Horizonte, de maneira a atender às determinações da Resolução nº 466/2012 do Conselho Nacional de Saúde. Para coleta de dados, reitera-se que todos os participantes assinaram o Termo de Consentimento Livre e Esclarecido antes dos questionários serem aplicados.

### Desenho, período e local do estudo

Trata-se de estudo observacional, analítico e transversal, com vistas a examinar a relação entre liderança *coaching* e fatores sociodemográficos e relacionados à profissão. O presente artigo seguiu as recomendações do *STrengthening the Reporting of OBservational studies in Epidemiology* para subsidiar a comunicação dos resultados evidenciados^(13)^. O cenário do estudo foi a Rede de Urgência e Emergência (RUE) de uma cidade da Região Metropolitana da capital do estado de Minas Gerais. Essa RUE é constituída pelo SAMU 192 e nove Unidades de Pronto Atendimento (UPAs). Participaram da pesquisa o SAMU 192 e seis UPAs da capital metropolitana de Minas Gerais. Três UPAs não foram incluídas por não responderem aos e-mails para realização da coleta de dados. A coleta de dados transcorreu entre julho e dezembro de 2023.

### População, amostra; critérios de inclusão e exclusão

O número total de enfermeiros nas unidades participantes da pesquisa, tanto no âmbito assistencial quanto no gerenciamento, totalizou 258 profissionais. Incluíram-se enfermeiros que estivessem exercendo o cargo há pelo menos seis meses, de maneira a garantir que o profissional já tivesse se ambientado e, assim, estivesse apto a analisar a prática da liderança. Excluíram-se os profissionais da categoria supracitada que estivessem em gozo de férias, em licença-prêmio ou em afastamento por motivos de saúde no período da coleta de dados.

Diante desses critérios, aponta-se que: 34 enfermeiros estavam em período de férias; dez estavam em gozo de licença médica; 39 profissionais não atendiam o tempo mínimo de seis meses na instituição; e 21 profissionais recusaram participar da pesquisa. Assim, 154 enfermeiros estavam efetivamente elegíveis para a pesquisa. A partir desse valor, aplicou-se o cálculo amostral, adotando-se o erro amostral de 8%, nível de confiança de 95% e uma distribuição da população mais homogênea (80/20), obtendo-se 61 participantes. Tratou-se de uma amostra por conveniência, e os pesquisadores optaram por entregar os questionários a todos os enfermeiros que trabalhavam no SAMU e nas seis UPAs no período da coleta. Ressalta-se que foram considerados como perda da amostra para o presente estudo os profissionais que, após duas tentativas, não foram localizados nos seus respectivos plantões e os profissionais que não devolveram os questionários preenchidos ou em branco.

### Instrumentos, coleta e análise dos dados

Foram utilizados dois questionários, sendo o primeiro construído pelos próprios pesquisadores, não sendo validado por juízes e correspondendo à caracterização dos participantes, como dados sociodemográficos e profissionais, cujas informações coletadas consistiram em: idade; sexo; tempo de formação; formação complementar; tipo de vínculo empregatício; tempo de atuação na unidade; possuir outro vínculo empregatício; e já ter assumido cargo de coordenação/chefia de enfermagem.

Aplicou-se a terceira parte do instrumento Questionário de Autopercepção do Enfermeiro no Exercício da Liderança (QUAPEEL), que contém itens relacionados às habilidades e atitudes exercidas pelos líderes na prática da liderança *coaching*, tendo em vista a autopercepção do profissional acerca da liderança *coaching*. Esse instrumento foi construído e validado no Brasil e, para a realização desta pesquisa, foi previamente solicitada e concedida a autorização da autora para utilização do instrumento^(6)^.

Esse questionário dispõe de 20 itens, subdivididos em quatro domínios: Comunicação (itens 1 a 5); Dar e receber *feedback* (itens 6 a 10); Dar e exercer influência (itens 11 a 15); e Apoiar a equipe para alcance de resultados (itens 16 a 20). Para todos os itens, há uma escala Likert disposta da seguinte maneira: nunca (1); raramente (2); nem sempre (3); quase sempre (4) e; sempre (5). O “não se aplica” (NA) compreende ao item que o profissional acredita não desenvolver, sendo atribuído, nesse sentido, o valor de zero. Dessa forma, o escore do instrumento geral varia entre 0 e 100, considerando-se que os valores mais próximos de 0 correspondem à menor percepção da prática de liderança *coaching,* e 100, à maior percepção da prática desse tipo de liderança^(6)^.

O grupo, constituído por um docente pesquisador e quatro alunas pesquisadoras, foi responsável por entregar os questionários aos sujeitos que consentissem participar da pesquisa, sendo, para tanto, realizado previamente o contato com os coordenadores administrativos e de enfermagem das unidades em questão para que fossem entregues os questionários aos enfermeiros pessoalmente, bem como realizado o agendamento do recolhimento dos instrumentos. Cabe apontar que, para realizar a entrega, foram realizadas três tentativas, em datas distintas, de maneira a encontrar os profissionais nos seus respectivos plantões. Após essas três tentativas sem sucesso, foi considerada como perda na população.

Os dados coletados por intermédio dos questionários foram duplamente digitados em planilhas e confrontados, assegurando-se, dessa forma, não haver erros de digitação, sendo, posteriormente, realizada a análise estatística descritiva e de correlação entre as variáveis. Para isso, empregou-se o programa estatístico R, versão 4.3.3, para o tratamento dos dados. As variáveis qualitativas foram resumidas em frequências simples e relativas, utilizando-se, para tanto, as porcentagens. Já as variáveis quantitativas foram organizadas por média, mediana, desvio padrão, primeiro e terceiro quartil e valor mínimo e máximo.

Inicialmente, foi utilizado o teste de normalidade Shapiro-Wilk para avaliação da distribuição das variáveis, ao passo que, para a análise de correlação entre as variáveis quantitativas, como idade, tempo de formação e tempo na instituição, e as variáveis da liderança *coaching,* foi aplicada a correlação de Pearson. Da mesma forma, os testes estatísticos Wilcoxon-Mann-Whitney e Kruskal-Wallis foram utilizados para analisar o nível de significância entre as variáveis de liderança *coaching* e as variáveis sociodemográficas e profissionais. Adotou-se o nível de significância de 5% para todos os testes estatísticos (α=0,05).

Complementarmente ao teste de Pearson, as associações entre variáveis presentes no estudo foram avaliadas conforme o tamanho da correlação, positiva ou negativamente, podendo, assim, apresentar: correlação muito alta (0,9 a 1,0); correlação alta (0,7 a 0,89); correlação moderada (0,40 a 0,69); correlação fraca (0,1 a 0,39); e correlação insignificante (0,0 a 0,3 ou 0,0 a -0,3)^(14)^.

## RESULTADOS

Ao final da coleta de dados, obteve-se um porcentual de 48,17% (n=79) de participantes. Convém destacar que os demais profissionais (n=75), ou seja, 53 deles, não devolveram os envelopes com os questionários e, após duas tentativas, 22 desses profissionais não foram localizados devido às trocas nos seus respectivos plantões.

Constatou-se que os enfermeiros, predominantemente, eram do gênero cis feminino (n=61; 77,22%), concursados, ou seja, regidos pelo regime estatutário (n=35; 44,30%), trabalhadores com outro vínculo empregatício (n=40; 50,63%) e enfermeiros assistenciais, pois nunca assumiram cargo de coordenação/chefia de enfermagem (n=49; 62,03%). Quanto à formação, um número considerável de profissionais realizou uma especialização (n=70; 88,61%); dez profissionais tinham mestrado; apenas um enfermeiro tinha titulação de doutor (1,27%); e pouco menos da metade possuía curso complementar à sua formação (n=37; 46,84%). Em relação às variáveis quantitativas relacionadas aos aspectos sociodemográficos ou ligadas à profissão, os enfermeiros apresentavam médias de 41,10 anos de idade (DP=8,12), 13,66 anos de formação em relação à data de conclusão da graduação (DP=7,32) e 5,95 anos de vínculo na unidade (DP=6,35). A Tabela 1 apresenta a distribuição dos escores das medidas da autopercepção dos enfermeiros acerca da prática de liderança *coaching* exercida pelos próprios enfermeiros nas unidades do APH.

**Tabela 1:** Escores da autopercepção dos enfermeiros exercida nas unidades de atendimento pré-hospitalar, Belo Horizonte, Minas Gerais, Brasil, 2023 (N=79)

Domínios	Média	Desvio padrão	Mediana	Mínimo	Máximo
Comunicação	22,47	2,25	23	17	25
Dar e receber *feedback*	21,72	2,60	22	13	25
Dar e exercer influência	20,85	3,23	21	8	25
Apoiar a equipe para alcance de resultados	18,85	4,80	20	5	25
Escore total do QUAPEEL	83,89	10,44	84	55	100

A Tabela 1 demonstra que os enfermeiros tiveram a autopercepção do exercício da liderança *coaching*, tendo em vista a média do escore total do QUAPEEL, que obteve o valor de 83,89 (DP=10,44), ou seja, a média da escala se aproxima mais de 100 do que de zero.

Quando realizadas as correlações da liderança *coaching* com o tempo de formação, tempo de atuação na unidade e idade, verifica-se que não há correlação (Tabela 2).

**Tabela 2:** Correlação entre as dimensões da prática da liderança *coaching* na autopercepção dos enfermeiros das unidades de atendimento pré-hospitalar e as variáveis quantitativas sociodemográficas e profissionais, como idade, tempo de formação e tempo na unidade, Belo Horizonte, Minas Gerais, Brasil, 2023 (N=79)

Domínios	Tempo de formação	Tempo na unidade	Idade
Comunicação	0,021^*^	-0,080	0,044
	0,856^**^	0,483	0,703
Dar e receber *feedback*	0,053	-0,121	-0,042
	0,641	0,289	0,716
Dar e exercer influência	0,097	-0,006	0,037
	0,397	0,962	0,746
Apoiar a equipe para alcance de resultados	-0,070	-0,201	-0,117
	0,543	0,076	0,304
Escore total do QUAPEEL	0,016	-0,141	-0,043
	0,891	0,214	0,704

* Correlação de Pearson;

** Valor de p.

A partir da Tabela 3, verificou-se que liderança *coaching* apresentou diferença estatisticamente significativa para o gênero cis masculino e a dimensão “Apoiar a equipe para alcance de resultados”, ou seja, os enfermeiros do gênero cis masculino têm maior percepção em promover o apoio à equipe (p=0,044); o profissional cujo tipo de vínculo é por tempo determinado; a dimensão “Comunicação” é exercida de forma mais presente (p=0,043); e não possuir outro vínculo empregatício influencia a percepção da dimensão “Comunicação” (p= < 0,001).

**Tabela 3:** Médias dos escores das dimensões e do escore total do Questionário de Autopercepção do Enfermeiro no Exercício da Liderança dos enfermeiros atuantes nas unidades de atendimento pré-hospitalar, conforme as variáveis qualitativas sociodemográficas e profissionais, e a correlação entre essas variáveis, Belo Horizonte, Minas Gerais, Brasil, 2023 (N=79)

		Comunicação	Dar e receber *feedback*	Dar e exercer influência	Apoiar a equipe para alcance de resultados	Escore total do Questionário de Autopercepção do Enfermeiro no Exercício da Liderança
Sexo	Feminino	22,28	21,61	20,62	18,38	82,89
	Masculino	23,11	22,11	21,61	20,44	87,28
Valor de *p* ^*^		0,070	0,436	0,304	0,044	0,086
Tipo de vínculo	Consolidação das Leis do Trabalho	22,17	21,33	20,11	18,83	82,44
	Estatutário	22,09	21,29	20,91	17,69	81,97
	Contrato por tempo determinado	23,19	22,58	21,27	20,42	87,46
Valor de *p* ^*^		0,043	0,102	0,629	0,083	0,101
Ter curso complementar	Não	22,60	21,86	21,21	19,07	84,74
	Sim	22,32	21,57	20,43	18,60	82,92
Valor de *p* ^*^		0,523	0,606	0,384	0,764	0,443
Especialização	Não	21,89	22,00	21,33	20,33	85,56
	Sim	22,54	21,69	20,79	18,66	83,67
Valor de *p* ^*^		0,638	0,969	0,810	0,670	0,613
Mestrado	Não	22,54	21,75	21,04	19,03	84,36
	Sim	22,00	21,50	19,50	17,60	80,60
Valor de *p* ^*^		0,271	0,959	0,317	0,245	0,309
Doutorado	Não	22,51	21,72	20,82	18,81	83,86
	Sim	19,00	22,00	23,00	22,00	86,00
Valor de *p* ^*^		0,141	0,930	0,480	0,441	0,930
Outro vínculo empregatício	Não	23,28	21,92	20,85	18,46	84,51
	Sim	21,68	21,53	20,85	19,23	83,28
Valor de *p* ^*^		< 0,001	0,523	0,650	0,887	0,533
Assumiu cargo de coordenação/chefia de enfermagem	Não	22,59	21,57	20,49	18,18	82,84
	Sim	22,27	21,97	21,43	19,93	85,60
Valor de *p* ^*^		0,377	0,879	0,368	0,205	0,256

* Wilcoxon-Mann;

** Whitney e Kruskal-Wallis.

## DISCUSSÃO

A liderança compreende um campo de estudo na enfermagem ao longo dos anos que explica, em parte, sua influência no ambiente profissional e resultados organizacionais, a exemplo do contexto da urgência e emergência^(2,3)^. As diversas teorias e modelos de liderança contribuem para compreensão, no âmbito do processo do trabalho da enfermagem, como as relações entre líderes e liderados ocorrem^(15)^. Os dados sociodemográficos e aqueles relacionados ao trabalho da presente pesquisa se assemelham aos de pesquisas que analisaram a mesma população e instrumento^(10,12)^ no contexto da urgência^(2)^. Nesse sentido, cabe destacar que o desempenho das lideranças na enfermagem sofre influência dos traços das gerações, pois à medida que a Geração Z ocupa o lugar na força de trabalho em enfermagem e nas chefias, tem-se observado aumento potencial para as mudanças e sucesso organizacionais, apoio ao trabalho colaborativo, facilidade na aprendizagem partilhada, e construção de relações de trabalho fortes e eficazes^(16)^.

Destaca-se que a liderança *coaching* foi autopercebida pelos enfermeiros do contexto pré-hospitalar móvel e fixo com média (83,89), ou seja, próxima ao valor de 100, também evidenciado no estudo pré-hospitalar do estado de Goiás (média de 80,91)^(2)^ e nas quatro pesquisas desenvolvidas em ambientes hospitalares, cujas médias do escore total do instrumento foram 90,67^(7)^, 84,90^(10)^, 84,41^(17)^ e 82,59^(11)^. Diante desse resultado, a liderança *coaching* figura uma competência potente para a *performance* no trabalho de enfermagem, tendo em vista que favorece o desenvolvimento de equipes e melhoria do desempenho profissional, especialmente no que diz respeito a auxiliar os funcionários a compreender melhor a si mesmos e suas capacidades^(8)^.

A comunicação é uma competência imprescindível para uma liderança bem-sucedida dos enfermeiros, em especial a liderança *coaching*, pois por intermédio de uma comunicação efetiva, o enfermeiro consegue compartilhar suas visões e influenciar sua equipe. Nesse espectro, entre os domínios do instrumento, a maior média encontrada foi a correspondente à comunicação, com a média de 22,47. Outros três estudos que aplicaram o QUAPEEL também identificaram a comunicação como a principal dimensão da liderança *coaching* ^(7,10,11)^. Corroborando esse achado, revisão integrativa sugere que os recursos investidos em habilidades de comunicação dos enfermeiros líderes trazem benefícios significativos em termos de satisfação da equipe, retenção de profissionais, qualidade do atendimento ao paciente e, finalmente, sucesso organizacional^(8,18)^.

Apoiar a equipe para o alcance dos resultados obteve o menor valor entre as médias das dimensões do instrumento utilizado na pesquisa (média de 18,85). Coadunando com esse achado, outros quatro estudos que analisaram a liderança *coaching* no contexto hospitalar e um no APH apresentaram a menor média nesse domínio, em comparação com os demais^(2,7,10,11,17)^. Isso ratifica que ainda é necessário, para as unidades de APH móvel e fixo, aprimorar as habilidades e atitudes dos enfermeiros para estímulo e apoio à equipe para o alcance de metas.

Ao considerar o cenário do presente estudo, fica evidente que o enfermeiro assume papel fundamental no gerenciamento da urgência e emergência, valendo-se de suas potencialidades, com destaque para a liderança e gestão da equipe de enfermagem^(3)^. Não foi identificada correlação entre as variáveis quantitativas do estudo e as dimensões e escore total do QUAPEEL. Semelhantemente, estudo desenvolvido no interior de São Paulo identificou também que as variáveis idade e o tempo de formação profissional dos respondentes não influenciaram a avaliação da prática da liderança exemplar^(4)^.

Em contrapartida a esses achados, foi identificada, em outro estudo, a correlação negativa entre “Dar e receber *feedback*” e “tempo de formação”, ou seja, à medida que o tempo de formação aumenta, menor a autopercepção dos enfermeiros quanto ao processo de *feedback*. Nesse mesmo estudo, houve também uma relação inversa entre o “Escore total da QUAPEEL”, da liderança *coaching,* e a variável “tempo de formação”, denotando, assim, que quanto maior o tempo de formação, menor a autopercepção da prática da liderança *coaching* ^(2)^. Nota-se que o tempo de formação propiciar ao líder maior facilidade na detecção das necessidades de desenvolvimento, bem como capacitação dos profissionais no sentido de transpor os desafios indissociáveis da atuação profissional, por intermédio de *feedbacks* que sejam significativos e contribuam para o alto desempenho da equipe e para o alcance das metas organizacionais^(8)^.

Apoiar a equipe para alcance de resultados apresentou diferença estatisticamente significativa para o gênero cis masculino (valor de p = 0,044). Sob esse aspecto, cabe apontar que a literatura sobre sociologia, psicologia e estudos de gênero frequentemente discute como a socialização de gênero influencia a assunção de papéis de liderança e apoio em ambientes de trabalho. Historicamente, os homens são incentivados nas organizações de saúde a assumirem papéis de liderança e apoio mais do que as mulheres, contribuindo, assim, para um olhar especial acerca de como homens se comportam e são percebidos no ambiente de enfermagem. Por sua vez, as mulheres na enfermagem podem enfrentar estereótipos que as colocam em posições somente de suporte e cuidado direto com o paciente, o que pode influenciar suas interações com a equipe^(19,20)^.

Revisão integrativa aponta que os homens na enfermagem recebem, em geral, tratamento preferencial na progressão na carreira e são mais aceitos por gestores, pacientes e outros profissionais de saúde nos processos de trabalho^(20)^. Assim, considerando-se a escassez crítica que afeta a profissão a nível mundial, é fundamental a promoção de ambientes de trabalho que estimulem a força de trabalho de enfermagem com maior diversidade, especialmente em termos de gênero, raça e etnia^(21)^.

A comunicação foi influenciada por variáveis qualitativas, como vínculo de emprego por contrato por tempo determinado e não possuir outro vínculo empregatício. Essas relações podem ser justificadas pelas transformações do mercado de trabalho da enfermagem, assim como a progressiva precarização dos vínculos trabalhistas no setor público que impactam os processos e as relações de trabalho e, consequentemente, os processos comunicativos das organizações de saúde^(22)^. A liderança *coaching* pode desempenhar um papel no desenvolvimento da eficácia da comunicação, resolução de conflitos promover um ambiente de trabalho saudável^(8)^.

Não houve diferença estatisticamente significativa entre as variáveis relacionadas à liderança *coaching* e ter assumido ou não cargo de coordenação/chefia de enfermagem. Considerando-se esse resultado, é válido apontar que um líder pode influenciar e guiar pessoas sem ter autoridade formal sobre elas (liderança informal). Soma-se a isso ao fato que a liderança *coaching* se baseia na construção de confiança e na criação de um ambiente de apoio e colaboração, enquanto um cargo de chefia frequentemente vem com autoridade formal e responsabilidade hierárquica^(8,23,24)^.

Ressalta-se apenas que, no presente estudo, não foi evidenciada uma relação entre as dimensões, assim como o escore total do QUAPEEL, e ter especialização, mestrado, doutorado ou curso complementar. A liderança *coaching* constitui uma competência indispensável para o alcance de um ambiente saudável de trabalho, o que resulta na segurança do paciente, elevada satisfação profissional e redução das taxas de rotatividade da equipe^(8)^. Nesse mesmo sentido, é válido citar estudo desenvolvido no contexto da urgência e emergência, no sul de Minas Gerais, o qual identificou que, na percepção dos enfermeiros, o conhecimento, a experiência profissional e a capacidade de se relacionar são cruciais para o sucesso profissional e para bons resultados nos atendimentos aos usuários dos serviços^(25)^.

Corroborando esse achado, estudo apontou que, para o desenvolvimento da liderança, são necessárias estratégias que englobem a busca por conhecimento (como os cursos de pós-graduação e formação complementar), aprimoramento de habilidades e adoção de atitudes positivas, constituindo, assim, elementos essenciais para uma *performance* adequada dos enfermeiros no exercício da sua prática profissional^(15)^. É oportuno destacar o grande desafio dos líderes de enfermagem diante do cenário atual no que diz respeito ao desenvolvimento da liderança, por intermédio do envolvimento, da empatia da equipe e da capacidade de influenciar e agir entusiasticamente^(26)^. Por fim, embora não evidenciada a relação entre a experiência em cargos de coordenação e chefia de enfermagem com os domínios da liderança *coaching*, revisão aponta que comportamentos de *coaching*, como *feedback* e comunicação aberta, podem influenciar a identificação organizacional pelo profissional e o bem-estar no local de trabalho^(8)^.

### Limitações do estudo

Configura-se como limitação do estudo o fato de não ter conseguido se valer das respostas de todos os enfermeiros das unidades investigadas em decorrência das trocas de plantões. Embora tenha se observado essa dificuldade, a magnitude e o caráter inovador do trabalho são assegurados pelo fato de conseguir analisar tanto o contexto do APH móvel, na qualidade do SAMU, quanto as UPAs. Outra limitação do estudo é a não validação do instrumento de caracterização dos participantes da pesquisa, mas esse fato não minimiza os achados da pesquisa.

### Contribuições para as áreas da saúde, enfermagem ou políticas públicas

Mesmo diante dessas limitações, o presente trabalho se torna relevante por ter implicações práticas para a gestão e para a organização dos serviços de APH móvel e fixo, principalmente no que tange ao gerenciamento da equipe de enfermagem acerca dos fatores que influenciam a liderança desses profissionais. Portanto, os gestores devem incentivar e promover condições adequadas para que os enfermeiros possam exercer legitimamente a sua liderança, sendo devidamente reconhecida.

## CONCLUSÕES

Por intermédio dos achados do estudo, os enfermeiros das unidades analisadas tiveram a autopercepção da liderança *coaching*. A dimensão “Comunicação” obteve a maior média diante das demais, o que demonstra a relevância desse item para prática da liderança do enfermeiro das unidades de pré-hospitalar móvel e fixo. Verificou-se que a liderança *coaching* sofreu influências de algumas variáveis sociodemográficas ou relacionadas à profissão. Constatou-se a correlação entre gênero cis masculino e a dimensão “Apoiar a equipe para o alcance dos resultados”, da mesma forma que a dimensão “Comunicação” se relacionou com as variáveis qualitativas “contrato por tempo determinado” e “não possuir outro vínculo empregatício”. Não foram encontradas diferenças e correlações estatisticamente significativas entre as dimensões e o escore total do QUAPEEL com as variáveis quantitativas do estudo. Os resultados do trabalho apontam para as possibilidades de melhorias, de novos caminhos para se liderar a equipe, de maneira a alcançar as metas estabelecidas no contexto pré-hospitalar móvel e fixo.

## Data Availability

Os dados da pesquisa estão disponíveis no corpo do artigo.
